# The Clinical Characteristic and Management of Patients with Nocardiosis in a Tertiary Hospital in China

**DOI:** 10.4014/jmb.2209.09034

**Published:** 2023-01-06

**Authors:** Peilin Liu, Zhiqian Wang, Zijuan Jian, Xuan Liu, Yanming Li, Qun Yan, Baiyun Zhong, Mengting Liao, Xianghui Liang, Wenen Liu

**Affiliations:** 1 Department of Clinical Laboratory, Xiangya Hospital, Central South University No.87 Xiangya Road, Kaifu district, Changsha 410008, Hunan, P.R. China; 2 National Clinical Research Center for Geriatric Disorders, Xiangya Hospital, P.R. China

**Keywords:** *Nocardia*, Nocardiosis, clinical characteristic, immunosuppressed patients, early diagnosis

## Abstract

Nocardiosis is an uncommon opportunistic bacterial infection which becomes a significant health problem due to its increasing incidence and high mortality rate. However, many nocardiosis patients are underdiagnosed by physicians. To summarize the clinical characteristics and management of nocardiosis would help with better diagnosis and prognosis of nocardiosis. This retrospective study was conducted based on the medical records of nocardiosis patients between January 2015 and December 2021 in a tertiary hospital in China. Overall, 44 nocardiosis patients with 54 specimens were included. The patients consisted of 26 males and 18 females with a mean age of 50.4 ± 13.2 years. Among 44 patients, 26 (59.1%) were previously given immunosuppressive therapy. Connective tissue diseases (CTDs) were the most common underlying disease (16/44). The most frequent infection sites were the lungs (17/44) and skin or soft tissues (8/44). Common symptoms included cough (23/44), expectoration (18/44), fever (15/44), and subcutaneous abscesses (15/44). Forty-five out of 54 specimens (83.3%) required over 48 hours of culture time for nocardiosis detection. Thirty-six patients were cured or improved, 5 patients were discharged from the hospital due to poor prognosis, and 1 patient died. The average diagnosis time of poor prognosis cases was 19.7 days, which was significantly longer than those of improved or cured patients (7.3 days). Immunosuppressed patients comprise a large part of nocardiosis cases, which is worth attention in clinical practice. Early diagnosis, specifically through prolonged cultivation time of specimen, could help achieve better prognosis of nocardiosis patients.

## Introduction

*Nocardia* is a genus Gram-positive bacterium that belongs to aerobic actinomycetes [1]. It is pervasive in soil, fresh air, saltwater, and dust [2]. *Nocardia* was first discovered in 1888 by veterinarian Edmond Nocard [3, 4]. Advances in molecular techniques, such as 16S rRNA gene sequencing and matrix-assisted laser desorption ionization time-of-flight spectrometry have helped to identify over 100 species of *Nocardia* [1, 4, 5]. Approximately one-third of *Nocardia* species have been recognized as human pathogens [6, 7].

Nocardiosis most frequently involves the lungs, central nervous system (CNS), and skin and can also affect the joints, kidneys, and other organs [8, 9]. Several studies have revealed that immunocompromised patients and recipients of solid organ transplants were more susceptible to nocardiosis, especially the disseminated nocardiosis [10-12].

In recent years, the incidence of nocardiosis has increased significantly, which may be associated with an increasing number of immunocompromised patients and advancements in laboratory detection methods, along with more attention from clinicians due to its high mortality rate ranging from 20% to 30% [13-15]. However, *Nocardia* is easily missed, delayed or misdiagnosed in the clinic, resulting in inappropriate empirical therapy and poor outcomes of patients. Because *Nocardia* is an infrequent and difficult-to-culture bacteria and the isolation and identification of it usually takes days to weeks from the clinical specimen [13]. Moreover, there are no specific clinical signs and symptoms for the diagnosis of nocardiosis [16]. Consequently, the diagnosis of nocardiosis requires high level of suspicious and experience of clinicians and microbiology laboratory. To develop the diagnosis, treatment and prognosis of nocardiosis, a comprehensive and detailed knowledge of *Nocardia* identification and the clinical characteristics of nocardiosis is urgently required. Most of the existing literatures on nocardiosis consist of case reports or small case series, and there have been few systematic analyses of nocardiosis. Marked geographical variability in *Nocardia* distribution has also been investigated, but in China, such research remains rare [17]. Here, we retrospectively summarized the clinical characteristics and management of nocardiosis, including demographic data, underlying diseases, clinical manifestations, radiological examinations, diagnosis, treatment and outcomes that occurred in the past seven years in a tertiary hospital in China. Our finding will provide a better understanding of nocardiosis and contribute experience for the early clinical diagnosis and treatment, improving the prognosis of patients.

## Materials and Methods

### Clinical Data Collection

Data on nocardiosis cases were collected from patients in a tertiary hospital in China from January 2015 to December 2021. Patients with nocardiosis were identified from the records of the clinical microbiology laboratory. Demographic data (including sex, age, and visit time), underlying diseases [connective tissue disease (CTD), hypertension, respiratory disease, and history of immunosuppressive therapy], clinical manifestations (fever, cough, expectoration, subcutaneous abscesses, shortness of breath, muscle soreness, asthenia, altered consciousness, and hemoptysis), laboratory tests [routine blood tests, hepatic and renal function, erythrocyte sedimentation rate, serum C-reactive protein (CRP), and procalcitonin (PCT)], radiological examinations [chest computed tomography (CT) and magnetic resonance imaging], diagnosis, treatment (antimicrobial and surgical management), and outcomes were retrospectively reviewed.

### Isolation and Identification of *Nocardia*

In this study, all *Nocardia* strains were isolated from clinical specimens. Different sample types were collected, including sputum, bronchial secretions, bronchoalveolar lavage, skin abscesses, abscess puncture fluid, wound secretion/pus, blood, cerebrospinal fluid (CSF), brain abscess drainage, ocular secretion, peritoneal effusion, joint fluid, and tissue blocks.

**Fig. 1:**
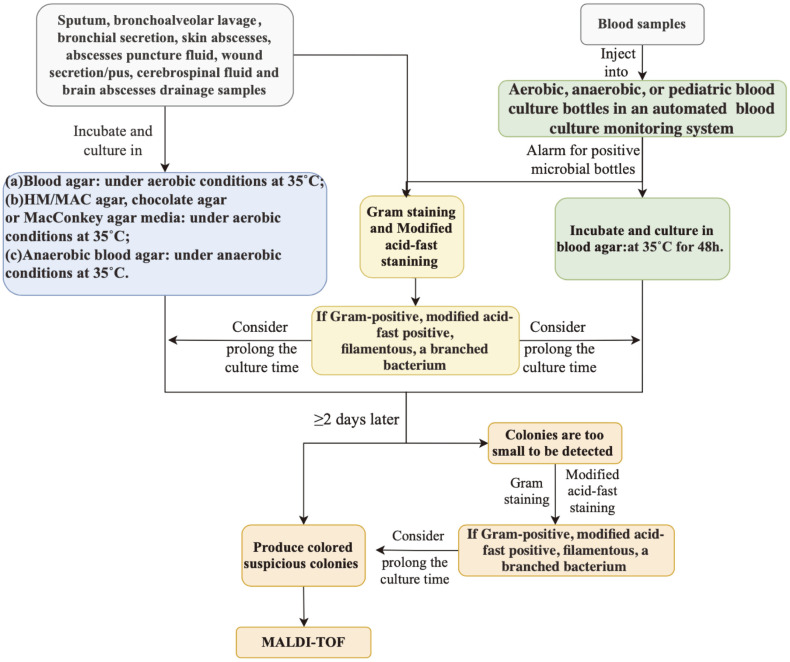
The specific microbiology examinations of common specimen. All *Nocardia* strains were isolated from different clinical specimens. The clinical specimens were prepared for smear microscopic examination, incubated, and cultured on different agar plates simultaneously. *Nocardia* isolates were identified using matrix-assisted laser desorption/ ionization time-of-flight mass spectrometry finally.

The clinical specimens were prepared for smear microscopic examination, incubated, and cultured on different agar plates simultaneously. For blood samples, it would inject into aerobic, anaerobic, or paediatric blood culture bottles and incubated in an automated blood culture monitoring system. routine culture plates were blood agar, HM/MAC agar, chocolate agar, MacConkey agar and anaerobic blood agar. If Gram-staining and modified acid-fast staining were positive and filamentous and a branched suspicious bacterium was found, microbiologists considered prolonging the conventional culture time (>2 days). *Nocardia* isolates were identified via matrix-assisted laser desorption/ionization time-of-flight mass spectrometry (MALDI-TOF-MS) using a MALDI Biotyper (Bruker Daltonics). A MALDI-TOF MS scores between 1.7 to 2.0 represented genus-level identification, while a score of higher or equal to 2.0 represented identification at species level. Anything less than 1.7 indicated unreliable identification. The specific microbiological examination procedures for the common specimens are shown in Fig. 1.

### Case Definition

A diagnosis of nocardiosis required at least one positive culture result for each sample, and all patients included in the analysis had complete information. Disseminated nocardiosis was defined as when two or more non-contiguous organs are clearly infected with *Nocardia* [18, 19].

### Statistical Analysis

Categorical variables were expressed as numbers and percentages. Continuous variables were presented as means ± standard deviation.

## Results

### Demographic Characteristic of Nocardiosis Cases

Forty-four patients (26 men, 18 women) with nocardiosis between January 2015 and December 2021 were included in this study. The mean age was 50.4 ± 13.2 years, ranging from 5 to 75 years old. Only one patient was younger than 20 years, and the majority (72.7%) were older than 45 years (Table 1).

Of the 44 patients, 36 had underlying diseases. The most common condition was CTD (16/44), followed by chronic kidney disease (10/44), hypertension (9/44), and diabetes (7/44) (Table 1). Nineteen patients had two or more underlying diseases.

Twenty-six patients (59.1%) were previously treated with glucocorticoids or immunosuppressive therapy, including intravenously administered methylprednisolone and oral prednisolone.

### Clinical Characteristic of Nocardiosis Patients

**Fig. 2:**
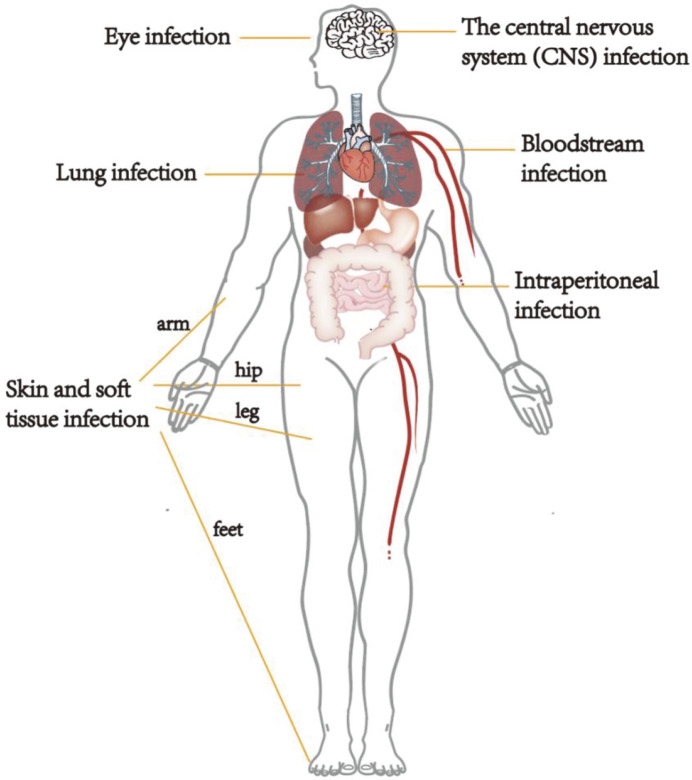
The distribution of infection sites. The most frequent infection sites were the lungs and skin and soft tissue. Twelve patients (27.3%) presented disseminated nocardiosis.

Seventeen patients (38.6%) only had pulmonary nocardiosis. Involvement of skin and soft tissue was found in 8 patients (18.2%), while the CNS was involved in 6 patients (13.6%), and 1 patient (2.3%) presented with an eye infection. Twelve patients (27.3%) presented with disseminated nocardiosis, including 6 patients with bloodstream infection, 4 patients with lung, skin, and soft tissue infection, and 2 patients with lung and intraperitoneal infections. The distribution of infection sites is shown in Fig. 2.

The clinical manifestations varied widely. Cough was noted in 23 patients (52.3%), expectoration in 18 patients (40.9%), fever in 15 patients (34.1%), subcutaneous abscesses in 15 patients (34.1%), shortness of breath in 10 patients (22.7%), headache in 7 patients (15.9%), muscle soreness in 6 patients (13.6%), asthenia in 6 patients (13.6%), and altered consciousness and hemoptysis in 4 patients each (10.3%) (Table 1).

### Isolates and *Nocardia* species Identification

*Nocardia* strains were isolated from 54 specimens, including sputum or bronchial secretions (*n* =16), bronchoalveolar lavage (*n* = 6), skin abscesses or abscess puncture fluid (*n* = 11), wound secretions or pus (*n* =7), blood (*n* = 6), CSF or brain abscess drainage (*n* =3), and others (*n* = 4). The average time taken for microbial detection was 4.8 days. Forty-five specimens exceeded the conventional culture time (>2 days) and prolonged the culture time. Twenty of these were found a Gram-positive, filamentous, or partially acid-fast branched bacterium suspected to be *Nocardia* from smear microscopic examination (Gram staining and modified acid-fast staining), so the microbiologists decided to extend their culture time. Twenty-four specimens required more than 4.8 days, and 11 specimens required more than 7 days.

**Fig. 3:**
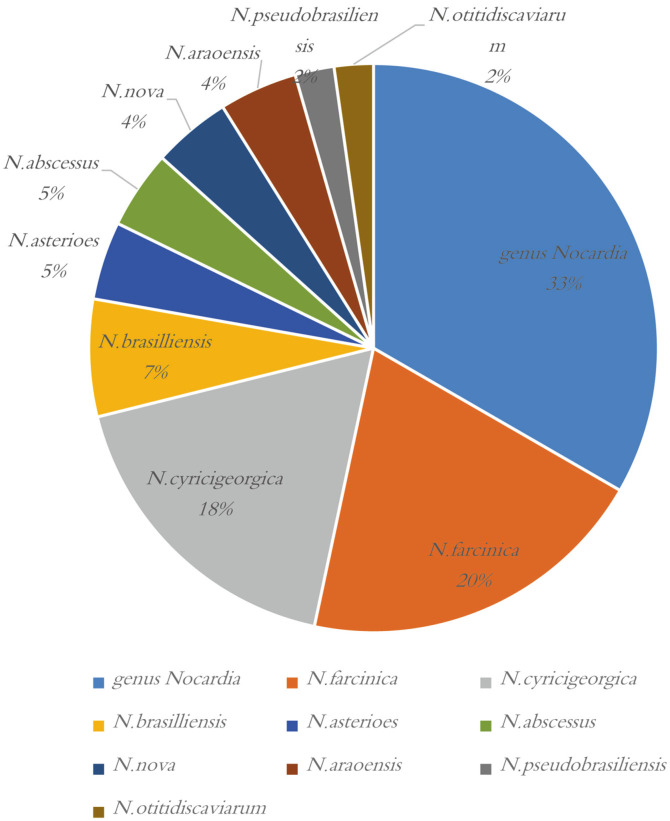
Distribution of *Nocardia* spp. Among the 44 *Nocardia* isolates, only 30 were identified beyond the genus level. The most common species isolated were *N. farcinica* (*n* = 9, 20.5%), *N. cyricigeorgica* (*n* = 8, 18.2%), *N. brasilliensis* (*n* = 3, 6.8%), *N. asterioes* (*n* = 2, 4.6%), *N. abscessus* (*n* = 2, 4.6%), *N. nova* (*n* = 2, 4.6%), *N. araoensis* (*n* = 2, 4.6%), *N. otitidiscaviarum* (*n* = 1, 2.3%), *N. pseudobrasiliensis* (*n* = 1, 2.3%), and 14 isolates could not be identified at the species level.

Among the 44 *Nocardia* isolates, only 30 were identified beyond the genus level. The most common species isolated were *N. farcinica* (*n* = 9, 20.5%), *N. cyricigeorgica* (*n* = 8, 18.2%), *N. brasilliensis* (*n* = 3, 6.8%), *N. asterioes* (*n* =2, 4.6%), *N. abscessus* (*n* =2, 4.6%), *N. nova* (*n* = 2, 4.6%), *N. araoensis* (*n* =2, 4.6%), *N. otitidiscaviarum* (n=1, 2.3%), and *N. pseudobrasiliensis* (*n* = 1, 2.3%). Fourteen isolates (*n* = 14, 31.8%) could not be identified at the species level (Fig. 3).

### Laboratory Tests and Radiological Examinations

Of the 44 patients, 26 patients (59.1%) showed elevated white cells, 30 patients (68.2%) showed elevated neutrophil proportion, and 34 patients (77.3%) showed decreased lymphocyte counts. Serum CRP levels were elevated in 26 patients (59.1%), and PCT levels were increased in 26 patients (59.1%). Twenty patients (45.5%) showed lower hemoglobin levels, and 7 patients (15.9%) showed elevated platelet counts. The level of serum album decreased in 32 patients (72.7%), 28 patients (63.6%) presented with an elevated ECR, and 9 patients (20.5%) had elevated serum creatinine levels(Table 1).

Bilateral involvement was detected in the chest CT images of 34 patients (77.3%), while 4 patients (9.1%) had unilateral involvement. All pulmonary nocardiosis cases showed abnormal chest CT findings. The most common radiological findings were nodules (38.6%), pleural effusion (15.9%), and patchy shadows (15.9%). Other imaging findings, such as cavities (11.4%), stripe-shape (11.4%), and mediastinal lymph node enlargement (9.1%) were relatively rare. Among the 6 CNS infection cases, 5 patients had intracranial space-occupying lesions, and 1 patient showed cerebral atrophy in magnetic resonance imaging.

### Management and Outcome

Twenty-nine patients were managed using only antimicrobial treatment, and 14 patients received both antimicrobial and surgical therapies. One patient had unclear medication.

Approximately 47.7% of patients received empirical antimicrobial therapy before the culture results were available, but they were rarely successful in those cases and clinicians usually changed the medication and eventually prescribed trimethoprim-sulfamethoxazole (TMP-SMX). Three patients (6.8%) were treated with TMP-SMX alone, and 28 patients (63.6%) had received other antibiotics in combination. The antimicrobial agents included oxazolidinones (linezolid), carbapenems (imipenem and meropenem), quinolones (moxifloxacin and levofloxacin), cephalosporins (ceftriaxone and cefoperazone-sulbactam), and aminoglycosides (amikacin). The remaining 12 patients were administered one or more antibiotics other than TMP-SMX.

Thirty-six patients were cured or improved, 5 were discharged from the hospital due to serious illness or poor prognosis, 2 outpatients were lost to follow-up, and 1 patient died. In addition, 2 patients experienced relapse, 1 of whom was cured, while the other patient died. In cases with poor prognosis (6 patients), all patients had underlying diseases, 5 patients used immunosuppressants, and 4 of which had CTDs. Moreover, 5 had received empirical antimicrobial therapy before the culture results were available.

The average diagnosis time of cases with poor prognosis was 19.7 days, while that of improved or cured patients was only 7.3 days. There was no difference in the average therapy duration between patients who had been cured/improved or had poor prognoses. The specific times for diagnosis, therapy, and disease duration are shown in Supplementary Material.

## Discussion

This is one of the largest contemporary single-center retrospective studies to describe the clinical characteristics and management of 44 patients with nocardiosis in China, to date. Nocardiosis mainly occurs in middle-aged and elderly men, and is frequently complicated by a series of underlying diseases [8, 20, 21]. Unlike other studies that reported chronic lung diseases as the most common underlying diseases, our findings suggest that immunocompromised patients, especially those with CTDs, can be vulnerable to nocardiosis [17, 22]. CTD patients on either glucocorticoids or immunosuppressive drugs were the most common nocardiosis cases at our institution, and the infection sites were predominantly in the lungs and skin. Consistently, a retrospective study from Israel showed that immunosuppressive drug therapy was positively correlated with nocardiosis [23].

Isolation and culture are the principal methods for diagnosis of nocardiosis, and longer culture time and smear microscopic examination improves detection sensitivity [24]. At present, isolation of *Nocardia* on common or selective media usually takes 2-7 days and some strains even need 2-4 weeks to generate available results, causing a delay in diagnosis[13, 16, 17, 25-27] . In our study, most specimens required extended culture time (>2 days). Hence, to avoid missing detection and increase the detection rates, clinicians should inform microbiologists of suspicious cases and require that more attention be paid to these specimens. Microbiologists should also consider prolonging the culture time and continue observation via the smear microscopic examination (Gram staining and modified acid-fast staining) on the other. A previous study of confirmed nocardiosis patients also demonstrated that Gram staining was a sensitive method for recognizing *Nocardia* in clinical specimens [28].

The distribution of *Nocardia* showed observable geographical variations. In our study, ten *Nocardia* species were identified among the 44 isolates. *N. farcinica* was the most common species, followed by *N. cyricigeorgica* and *N.brasilliensis*, which agreed with a recent study in China [15]. In other case series, the most frequently isolated *Nocardia* were diverse, including *N. cyricigeorgica* in Australia [17] and Japan [29], *N. brasiliensis* in Taiwan [18], and *N. nova* in the United States [30]. Moreover, a French retrospective analysis suggested that distributions may change over time, which has been reported for the increasing *N. farcinica* proportion from 2010 (13%) to 2014 (27.6%) [31]. Therefore, it’s meaningful to monitor the temporal and spatial changes in *Nocardia* distribution.

Clinical symptoms, radiological findings and inflammatory parameters (including leukocytes, neutrophils, CRP, and PCT) play an important role in the diagnosis of nocardiosis but lack specificity [28, 32]. In this study, the lungs and skin were the most common infection sites. Respiratory symptoms, detailed skin examination, and chest CT could be beneficial to clinicians in identifying the infection sites [8]. CNS infection could present with brain abscesses, and the frontal and parietal regions were commonly affected.

Empirical antimicrobial treatments before definite diagnosis do not work for all nocardiosis cases, and appropriate and personalized treatment after bacterial culture is recommended. In our study, approximately half of the cases recieved empirical antimicrobial therapy before the culture results were available, but they were rarely successful, and clinicians usually changed the medication and eventually prescribed TMP-SMX.

TMP-SMX is the drug of first choice for nocardiosis, and most *Nocardia* spp. are susceptible to it [29, 33]. For TMP-SMX-resistant, disseminated, or severe nocardiosis, a combination of TMP-SMX with other antibiotics is recommended [28]. Currently increased resistance towards TMP-SMX has been observed. One retrospective evaluation study in the USA from 1995 to 2004 showed a TMP-SMX resistance rate of 42% [30]. Another report from Spain in 2011 found that 16.1% cases were resistance to TMP-SMX [34]. Therefore, according to the Sanford Guide to Antimicrobial Therapy 2018 [35], TMP-SMX with imipenem or amikacin should be considered in disseminated nocardiosis patients. Linezolid is also an appealing alternative, because it has high bioavailability and most *Nocardia* show susceptibility [36]. Other alternative treatment options include minocycline, amikacin, and meropenem [19]. In terms of treatment, 3 months is recommended for immunocompetent patients, at least 6 months for immune-compromised patients, and at least a year for patients with CNS involvement [7]. It has been reported that longer therapy duration may be preferable to prevent recurrence [37]. In our study, nocardiosis management generally agreed with was the above therapies and most patients cured.

Earlier diagnosis and administration are beneficial for the prognosis, so clinicians’ awareness of nocardiosis should be improved to accelerate the process of diagnosis [33]. In cases with poor prognosis (6 patients), the clinicians did not suspect that the patients’ abnormal clinical characteristics were caused by *Nocardia* in the early stage. The average diagnosis time for patients with poor prognoses were much longer than those for the cured or improved patients, which caused delays in treatment of patients, likely led to initial noneffective empirical treatments and affected the prognosis.

Our study had some limitations. First, it was a single-center study, and the clinical characteristics are regional-limited; multi-center studies would improve the reliability of the data. Moreover, the sample size is small, thus a larger sample size and additional research data are needed to further support these conclusions. Second, as this was a retrospective study, some information, such as outpatient history, was incomplete. Third, we only used the MALDI-TOF-MS to identify the *Nocardia* isolates, and they were not verified by purified PCR product and sequencing the full length of the 16S RNA gene. Also, antimicrobial susceptibility tests were not performed in this study.

In conclusion, earlier diagnosis and administration are beneficial for improving the prognosis of nocardiosis. Attention should be paid to immunocompromised patients and nocardiosis should be considered. Prolonged cultivation time helps with nocardia detection. Improving diagnostic efficiency at early stage is of particular importance to achieve favorable prognosis in nocardiosis.

## Supplemental Materials

Supplementary data for this paper are available on-line only at http://jmb.or.kr.

## Figures and Tables

**Table 1:** The demographic and clinical characteristics of patients with nocardiosis cases.

Variables	Nocardiosis cases (*n* = 44)
Age(years), mean ± SD (range)	50.4 ± 13.2
Sex	
Male	26 (59.1%)
Female	18 (40.9%)
Underlying diseases	33 (75.0%)
CTD	16 (36.3%)
Chronic kidney disease	10 (22.7%)
Hypertension	9 (20.5%)
Diabetes	7 (15.9%)
Respiratory disease	5 (11.4%)
Viral hepatitis B	5 (11.4%)
CHD	3 (6.8%)
Leukemia	2 (4.5%)
Glucocorticoid or immunosuppressive therapy	26 (59.1%)
Infection sites	
Lung	17 (38.6%)
Skin and soft tissue	8 (18.2%)
CNS	6 (13.6%)
Bloodstream	6 (13.6%)
Lung, skin and soft tissue	4 (9.1%)
Lung and intraperitoneal	2 (4.5%)
Eye	1 (2.3%)
Clinical characteristics	44 (100%)
Cough	23 (52.3%)
Expectoration	18 (40.9%)
Fever	15 (34.1%)
Subcutaneous abscesses	15 (34.1%)
Shortness of breath	10 (22.7%)
Headache	7 (15.9%)
Muscle soreness	6 (13.6%)
Asthenia	6 (13.6%)
Altered consciousness	4 (10.3%)
Hemoptysis	4 (10.3%)
Chest pain	4 (10.3%)
Laboratory tests	
Elevated white cells	26 (59.1%)
Elevated neutrophil proportion	30(68.2%)
Decreased lymphocyte counts	34 (77.3%)
Decreased hemoglobin levels	20 (45.5%)
Elevated platelet levels	7 (15.9%)
Decreased serum album levels	32 (72.7%)
Elevated ECR	28 (63.6%)
Elevated serum creatinine	9 (20.5%)
Elevated PCT levels	26 (59.1%)
Elevated CRP levels	26 (59.1%)
Radiological examinations	
Bilateral involvement	34 (77.3%)
Unilateral involvement	4 (9.1%)
Management	
Only antimicrobial treatment	29 (65.9%)
Antimicrobial and surgical thrapy	14 (31.8%)
Outcome	
Cured or improved	36 (81.8%)
Unilateral involvement	4 (9.1%)
Management	
Only antimicrobial treatment	29 (65.9%)
Antimicrobial and surgical thrapy	14 (31.8%)
Outcome	
Cured or improved	36 (81.8%)
Poor prognosis	5 (11.4%)
Lost follow-up	2 (4.5%)
Died	1 (2.3%)

Abbreviations: CTD: Connective Tissue disease; CHD: Coronary heart disease; CNS: central nervous system
