# Supplementary material for: The Clinical Characteristic and Management of Patients with Nocardiosis in a Tertiary Hospital in China
Source: J Microbiol Biotechnol. 2023 Jan 6;33(5):574–81. doi: 10.4014/jmb.2209.09034 (PMC10236171; doi:10.4014/jmb.2209.09034)
Supplement: Supplementary file 1 [file jmb-33-5-574-supple.pdf]

J. Microbiol. Biotechnol.
https://doi.org/10.4014/jmb.2209.09034

Supplementary Tables

The Clinical Characteristic, Examination and Management of 44 patients with nocardiosis

a. The clinical characteristic and management of 44 patients with nocardiosis

Patient	Age/Sex	Underlying disease	Infection sites	Symptoms	Specimen type	Nocardia specie	Treatment （including surgical and antimicrobial management）	Time to diagnosis （day）	Therapy duration （day）	Disease duration (day)	Outcome
1	75/F	Non	Skin	Subcutaneous abscesses	Tissue block	Nocardia spp.	Surgical excision, TMP-SMX for 7 months	13	210	-	Cured
2	5/M	Trauma	Eye	Difficulty in eye open, ocular pain, tears	Vitreous tapping fluid	Nocardia spp.	Vitrectomy, CAZ+TOB eye drops for 5 days (continued medication post-discharge)	6	7+	10	Improved
3	29/F	ALL treated with MP, Hematopoietic stem cell transplant (1 years prior)	Lung	Shortness of breath, cough, expectoration	Bronchial secretion, sputum	Nocardia spp.	Before:SCF+VCZ(40d)→PIP-TAZ+POS(12d)→AMB+IMP(13d): no clinical improvement After: TMP-SMX+ LZD +POS (10d)	66	75	165	Worsened
4	64/M	Lung cancer	Lung	Fever, cough, expectoration	Sputum	N.otitidiscaviarum	IMP+CAZ(4d)	6	4	-	Lost to follow up
5	29/M	Chronic kidney disease treated with immunosuppressant, HBV	Brain	Intermittent convulsions, altered consciousness， nausea and vomiting	Puncture fluid	N.farcinica	Excision of intracranial abscess, CRO(7d) →VA(10d) (Continued medication post-discharge)	20	19+	139	Improved
6	54/M	AOSD treated with MP	Blood	Fever, shortness of breath,	Blood	N.farcinica	MEM+VCZ (1d)	6	1	300	Worsened

			stream infection	asthenia, sore throat, altered consciousness, muscle soreness, skin rash			（Treated several times in other hospital: no clinical improvement）				
7	67/F	Chronic bronchitis	Lung	Cough, expectoration, hemoptysis	bronchoalveolar lavage	N.araoensis	PIP-TAZ(5d) (Continued medication post-discharge)	9	5+	26	Improved
8	38/F	ALL treated with MP, hematopoietic stem cell transplant (3 years prior)	Blood stream infection, kidney	Fever, cough, expectoration, headache, lumbar back pain	Blood, puncture fluid	Nocardia spp.	Abscess drainage, Before:MEM+Cefoselis(4d) →IMP+VA+VCZ(2d)→CAS+TGC(8d) After:TMP-SMX+TGC+MXF(19d) (Continued medication post-discharge)	11	33+	93	Improved
9	37/M	ANCA-associated vasculitis treated with Prednisolone, renal dysfunction, Hypertension	Skin, lung	subcutaneous abscesses	Abscess puncture fluid, sputum,	N.brasilliensis	Before: MEM+VCZ (3d): no clinical improvement After: TMP-SMX+CRO+ AK (24d) (Continued medication post-discharge)	4	9+	29	Improved
10	28/M	DM treated with Prednisolone, Interstitial pneumonia	Skin, lung	Asthenia, skin redness, swelling and ulceration	Wound secretion	N.farcinica	Before:LEV+VCZ(5d) After:TMP-SMX+IMP(14d)→TMP-SMX(14d) (Continued medication post-discharge)	4	33+	93	Improved
11	56/M	CHD, hypertension, gout treated with MP, kidney stones	Lung	Shortness of breath, expectoration, cough	Sputum	N.brasilliensis	Before: MEM(3d),After:TMP-SMX+ IMP(14d): caused nausea and vomiting Changed to IMP +DOX(6d) (Continued medication post-discharge)	3	18+	78	Improved
12	56/M	Non	Lung	Cough, expectoration	Bronchial secretion	N.farcinica	FOX (15d) (Continued medication post-discharge)	6	15+	1080	Improved

13	53/M	MSA, Esophagitis	Brain	Headache, fever, nausea, vomiting, limb dyskinesia, speech disorder,	Puncture fluid, CSF	Nocardia spp.	Abscess drainage<br>Before: CRO+VA+MEM(3d)<br>After: TMP-SMX+LZD(15d)<br>(Continued medication post-discharge)	2	18+	21	Improved
14	57/F	Hypertension, RP with Prednisone	Lung	Fever, cough, expectoration, soreness of muscle	Lung tissue	N.nova	Before: MXF(5d)<br>After: TMP-SMX+CRO (9d): caused nausea and vomiting and no clinical improvement<br>TMP-SMX+ IMP+VCZ (16d)<br>(Continued medication post-discharge)	12	42+	62	Improved
15	56/F	ANCA-associated renal vasculitis treated with MP, Chronic kidney injury	Skin	Fever, asthenia, muscle soreness, subcutaneous abscesses，	Skin abscesses	N.cyricigeorgica	Before: LEV(3d)<br>After:TMP-SMX+IMP+VCZ (12d): caused nausea and vomiting→ DOX + MEM +CAS(16d)<br>(Continued medication post-discharge)	7	31+	51	Improved
16	58/M	ANCA-associated vasculitis treated with MP, Diabetes, histories of soft tissue abscesses	Skin, lung	Subcutaneous abscesses, cough, expectoration, hemoptysis	Skin abscesses	Nocardia spp.	Abscess drainage<br>Before: VCZ+MXF(3d)<br>After: TMP-SMX+MXF(14d)<br>(Continued medication post-discharge)	4	17+	30	Improved
17	55/M	HBV, Pemphigus treated with prednisolone acetate	Lung	Fever, headache, chill, cough	Sputum	Nocardia spp.	Cefoselis+AZT(12d)<br>(Continued medication post-discharge)	5	12+	32	Improved
18	52/F	Pulmonary nodules	Lung	Hemoptysis	Bronchoalveolar lavage	N.araoensis	RPT+INH+PZA(12d)<br>(Continued medication post-discharge)	7	12+	37	Improved
19	71/M	ANCA-associated	Skin, lung,	Fever, subcutaneous	Blood, Skin	N.farcinica	Before: MXF(3d)	4	17+	257	Improved

		vasculitis treated with MP Sodium Succinate, COPD，Diabetes	bloodstream infection	abscesses, soreness of muscle	abscesses		After: LZD+TMP-SMX(10d)→ LZD+ TMP-SMX+AMC(2d) (Continued medication post-discharge)				
20	54/F	Diabetes	Skin	Fever, subcutaneous abscesses	Wound secretion	N.cyricigeorgica	Abscess drainage FOX(4d) → PM(7d)→ Cefoselis (7d)→ KZ(9d)→ CTX(5d)→MOX(6d)	5	76	81	Cured
21	35/M	HBV	Lung	Cough, fever, shortness of breath	Sputum	Nocardia spp.	Before: MEM+TGC (2d) After: TMP-SMX(1d) （Treated several times in other hospital:no clinical improvement）	5	2	31	Worsened
22	69/M	Hypertension	Skin	Skin redness, swelling and ulceration, right side body weakness	Skin abscesses	N.brasilliensis	Before: TMP-SMX+AMC(4d)； After: TMP-SMX+MH(3d) (Continued medication post-discharge)	4	7+	34	Improved
23	47/M	Hypertension, Chronic nephritis syndrome treated with Prednisolone	Bloodstream infection, skin, lung	Fever, asthenia, expectoration, soreness of muscle, chest pain, cough, Subcutaneous abscesses	Blood, Skin abscesses, Bronchial secretion	N.farcinica	Before:MEM(1d) After:TMP-SMX+MEM(3d)→TMP-SMX+LEV(13d) (Continued medication post-discharge)	4	17+	57	Improved
24	43/M	Nephrotic syndrome treated with MP, Diabetes, bilateral renal cysts	Skin, lung	Subcutaneous abscesses	Wound secretion	N.asterioes	Before: MOX+ AMB(10d) After: TMP-SMX(5d) (Continued medication post-discharge)	7	15+	55	Improved
25	73/M	Hypertension, Nephrotic	Skin	Skin redness and swelling	Wound secretion	N.abscessus	TMP-SMX+DOX(7d)	4	10+	18	Improved

		syndrome treated with prednisone					(Continued medication post-discharge)				
26	66/F	Sjögren syndrome treated with MP,	Lung	Joint pain，Subcutaneous abscesses	sputum	N.cyricigeorgica	Before: Cefuroxime +LEV(12d), After: TMP-SMX (4d): caused nausea and vomiting→AK+LZD(5d)	19	21	41	Worsened
27	55/M	RA treated with prednisone, CHD, diabetes, hypertension	Lung	Cough, expectoration, shortness of breath	Bronchial secretion, sputum	N.cyricigeorgica	Before: PIP-TAZ+LZD(1d) After:TMP-SMX+IMP+LZD(3d) (Continued medication post-discharge)	4	4+	13	Improved
28	44/M	Non	Brain	Headache, cough, chest pain	Brain abscess	Nocardia spp.	Parietal foci resection Before:Cefoselis+MEM(7d) After: TMP-SMX+PIP-TAZ(7d) (Continued medication post-discharge)	8	16+	76	Improved
29	33/M	SLE treated with MP	Brain	Fever, altered consciousness	CSF	N.farcinica	CRO+FLUCZ(5d)→PIP-TAZ(5d) →MEM(4d)：no clinical improvement （Treated several times in other hospital:no clinical improvement）	16	15	45	Worsened
30	59/F	CTD treated with MP, hypertension	Lung, intraperitoneal	Lower extremity swelling, Abdominal pain	Pus	Nocardia spp.	Biopsy of the masses， Before: LEV(19d) After: TMP-SMX(7d) (Continued medication post-discharge)	26	34+	304	Improved
31	56/F	CHD	Lung	Cough, shortness of breath	Bronchoalveolar lavage	Nocardia spp.	Before: Nemonoxacin(12d) After: TMP-SMX(30d)	11	27	385	Improved

							(Continued medication post-discharge)				
32	52/F	DM treated with MP,	Eye, Lung, Blood strea m infection	Cough, expectoration, shortness of breath, Alteration of vision	Eye secretion，blood	N.cyricigeorgica.	Vitrectomy, First hospitalization: CRO+TMP-SMX (120d) Second hospitalization:PIP-TAZ + TMP-SMX +VCZ (1d)	6	108	120	Relapse and died
33	54/F	SLE treated with MP, Renal dysfunction	Skin	Cough, generalized aches, Subcutaneous abscesses	Skin abscesses	Nocardia spp.	Abscess drainage Before:MOX(1d)→VCZ+SCF (6d) After:TMP-SMX+DOX for 14 days (Continued medication post-discharge)	9	21+	51	Improved
34	53/M	Non	Lung	Cough, expectoration	Bronchial secretion	N.cyricigeorgica.	TMP-SMX for about 1 years	7	330	330	Cured
35	44/M	Non	Brain	Headache	Pus	N.asterioes	Parietal foci resection TMP-SMX (50d) (Continued medication post-discharge)	3	60+	70	Improved
36	45/F	Non	Lung	Cough, expectoration, and shortness of breath	Bronchial secretion	N.nova	PIP-TAZ+ LEV (7d) (Continued medication post-discharge)	3	7+	73	Improved
37	52/M	Nephritis treated with MP，HBV，diabetes	Lung, blood stream infection	Fever, cough, expectoration, chest pain, asthenia, headache	Blood	N.farcinica	Before: MEM+DOX(6d) After: TMP-SMX(8d) (Continued medication post-discharge)	7	16+	76	Improved
38	49/M	RA treated with prednisolone acetate	Skin	Subcutaneous abscesses	Pus	N.abscessus	Abscess drainage Before: TMP-SMX+LZD(10d) (Continued medication post-discharge)	4	23	164	Improved
39	50/F	Non	Lung	Cough，expectoration	Bronchoalveolar	Nocardia spp.	DOX+MXF(70d)	11	89+	529	Improved

					lavage		(Continued medication post-discharge)				
40	50/F	Nephritis treated with MP，Diabetes，Trauma	Brain	Headache, vomiting	Abscess puncture fluid	N.farcinica	Brain abscess drainage<br>Before:VA(1d)→MEM+LZD(2d)<br>After:TMP-SMX+IPM(3d)→TMP-SMX+AK+IMP(7d)<br>(Continued medication post-discharge)	8	9+	30	Improved
41	56/M	Hypertension	Lung, intraperitoneal	Cough，expectoration, chest pain	Bronchial secretion, Sputum	Nocardia spp.	First hospitalization: IMP (7d)→PIP-TAZ(1d)<br>Second hospitalization：TMP-SMX + CRO + AK(9d)(continued medication post-discharge)	5	19+	199	Relapse and cured
42	51/M	RA treated with MP	Skin	Lower extremity swelling, Subcutaneous abscesses	Joint fluid	N.pseudobrasiliensi	-	5	4	35	Lost to follow
43	47/F	Non	Lung	Cough，expectoration, Hemoptysis, fever, chest pain, shortness of breath	Bronchoalveolar lavage	N.cyricigeorgica.	After：TMP-SMX+LEV(14d)→AML+TMP-SMX for 46 days	12	62	3650	Cured
44	41/F	SLE treated with MP，HBV	Lung	Fever, cough，expectoration, joint aches	Bronchoalveolar lavage	N.cyricigeorgica.	Before: MOF(5d)<br>After: TMP-SMX(2d)<br>(Continued medication post-discharge)	4	7+	67	Improved

Abbreviations: M:Male F:Female; TMP-SMX: trimethoprim-sulfamethoxazole; CAZ: ceftazidime; TOB: tobramycin; MP: methylprednisolone; ALL: acute lymphoblastic leukemia; SCF: cefoperazone-sulbactam; VCZ:Voriconazole; PIP-TAZ: piperacillin-tazobactam; AMB: amphotericin B;IMP: imipenem-cilastatin; POS: posaconazole; LZD: Linezolid; CRO:Ceftriaxone; VA: vancomycin; MEM:meropenem; TGC: Tigecycline; CAS:caspofungin; MXF:moxifloxacin; AK:amikacin; LEV:levofloxacin; DOX:doxycycline; FOX:Cefoxitin; AZT:aztreonam; RPT:rifapentine; INH:isoniazid; PZA:pyrazinamide; AMC:amoxicillin–clavulanate; PM:cefepime; KZ:cefazolin; CTX:cefotaxime; MOX:moxalactam; MH:minocycline; FLUCZ:fluconazole; IPM:imipenem; AML:amoxicillin; AOSD: adult-onset Stills disease; DM:dermatomyositis; CHD: coronary heart disease; RA:rheumatoid arthritis; MSA: mixed sleep apnea; RP: relapsing polychondritis COPD: chronic obstructive pulmonary disease; SLE: systemic lupus erythematosus; CTD = connective tissue disease

The later words after "Before" means the antibiotic treatment prior to positive culture results. The later words after "After" means the antibiotic treatment follow to the positive culture results.

The “+” means continuing therapy post-discharge. The “Time to diagnosis” ranges from hospitalization to clin ical diagnosis. The “Disease duration” ranges from the onset of clinical symptoms to symptoms improvement or abandon treatment.

b. The examination of 44 patients with nocardiosis.

Patient	WBC (10^9/L)	NEUT (%)	CRP	PCT	Patient	WBC (10^9/L)	NEUT (%)	CRP	PCT
1	4.1	36.8	-	-	23	16.6	88.2	78.6	0.26
2	9.3	77.5	-	-	24	12.1	79.3	-	0.053
3	9.5	90.4	230	1.97	25	14.8	79.9	73.2	0.17
4	17.4	88.8	317	11.58	26	9.7	84.5	19.0	0.21
5	11.6	86.4	4.75	0.07	27	9.4	95.1	398.0	41.44
6	1.2	96.8	320	0.57	28	9.9	74.6	-	<0.05
7	3.8	55.1	3.66	<0.05	29	18.5	95.2	98.81	2.26
8	11.9	75.2	121	1.44	30	15.6	91.1	36.7	-
9	14.2	78.7	76.0	1.40	31	8.9	64.8	-	-
10	12.2	87.7	40.8	<0.05	32	9.6	90.4	18.9	1.69
11	14.6	79.1	198	1.58	33	13.8	95.6	225	1.48
12	7.9	74.5	-	-	34	6.4	62.3	-	-
13	14.5	87.0	-	-	35	17.1	90.0	-	-
14	20.5	94.3	277	2.82	36	10.6	61.4	4.0	<0.05
15	5.4	90.7	119	0.44	37	12.1	89.2	354	16.43
16	8.1	89.4	93.2	0.27	38	17.8	94.0	78.9	0.63
17	15.8	87.3	154	0.096	39	5.0	64.6	-	-
18	4.0	72.2	-	-	40	19.6	94.7	74.0	0.42
19	23.0	95.9	70.1	0.30	41	34.9	90.3	169.0	0.86
20	9.0	59.0	75.39	0.064	42	6.8	80.5	262.0	-
21	0.2	59.1	-	106.4	43	7.8	68.8	-	-
22	10.0	82.0	2.84	<0.05	44	8.3	71.6	-	-

Abbreviations:

WBC: White blood cell; NEUT: neutrophil; CRP: C-reactive protein; PCT: procalcitonin
